# Pneumopéritoine spontané idiopathique: à propos d’une observation

**DOI:** 10.11604/pamj.2020.35.76.11193

**Published:** 2020-03-16

**Authors:** Kpatékana Simlawo, Fousséni Alassani, Boyodi Tchangaï, Damigou Mawuli Sambiani

**Affiliations:** 1 Service de Chirurgie Générale du Centre Hospitalier Régional Lomé-Commune, Lomé, Togo; 2 Service de Chirurgie Viscérale du Centre Hospitalier Universitaire Sylvanus Olympio, Lomé, Togo

**Keywords:** Pneumopéritoine spontané, tomodensitométrie, traitement non opératoire, Spontaneous pneumoperitoneum, CT scan, non-operative treatment

## Abstract

Le pneumopéritoine résulte, dans la majorité des cas, d'une perforation du tractus gastro-intestinal dont le traitement est habituellement chirurgical. Son caractère spontané est rare, avec dans ce cas, une présentation inhabituelle qui met le chirurgien devant un dilemme diagnostic et thérapeutique. Nous rapportons un cas de pneumopéritoine spontané chez un sujet de 77 ans ayant bien évolué sous traitement non opératoire. Nous discutons des modalités de prise en charge de cette entité peu commune.

## Abstract (en)

Pneumoperitoneum, in the majority of cases, is caused by a perforation of the gastrointestinal tract whose treatment is usually surgical. It rarely occurs spontaneously. This study reports an unusual presentation of pneumoperitoneum which has put the surgeon in a massive diagnostic and therapeutic dilemma. We report the case of a 77-year old patient with spontaneous pneumoperitoneum evolving during non-operative treatment. We here discuss the management procedure for this uncommon disease.

## Introduction

Le pneumopéritoine traduit généralement la perforation d'un organe creux et nécessite dans la règle une sanction chirurgicale pour réduire la contamination péritonéale [1]. Dans de rares cas, il se produit en l'absence de toute perforation et est qualifié de spontané [2]. Le chirurgien est alors devant un dilemme diagnostique et thérapeutique: comment confirmer le caractère spontané et quel traitement proposer. Nous rapportons un cas de pneumopéritoine spontané idiopathique (PSI) chez un sujet âgé ayant bien évolué sous traitement conservateur et discutons des modalités de prise en charge de cette entité peu commune.

## Patient et observation

**Figure 1:**
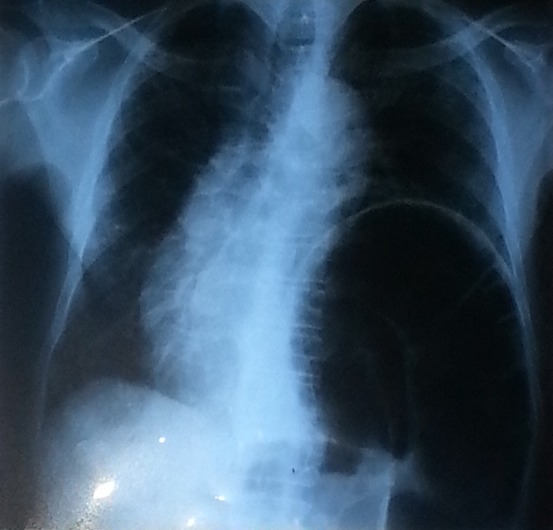
Radiographie de thorax de face complétant l’abdomen sans préparation montrant l’ascension de la coupole diaphragmatique gauche avec l’image en arceau et la dextrocardie

**Figure 2:**
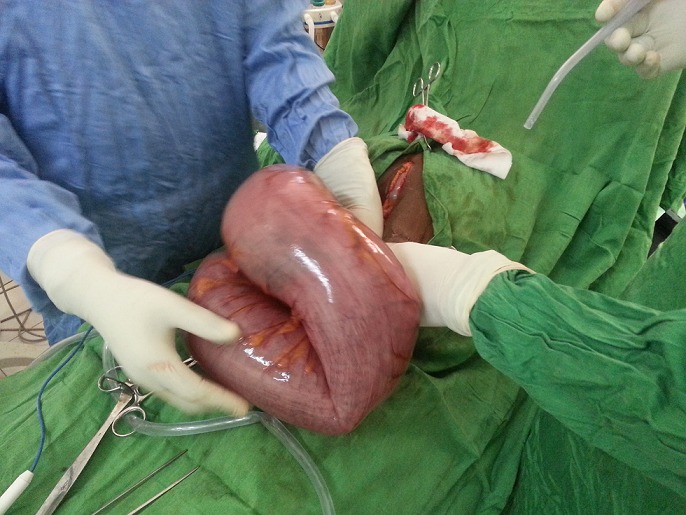
Image peropératoire du volvulus du côlon sigmoïde

**Figure 3:**
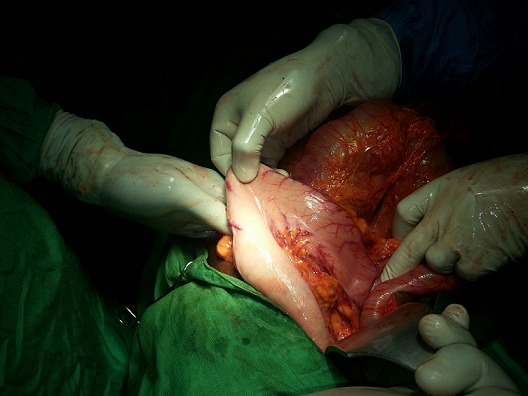
Image de l’estomac tracté de son ascension sous la coupole diaphragmatique gauche

**Figure 4:**
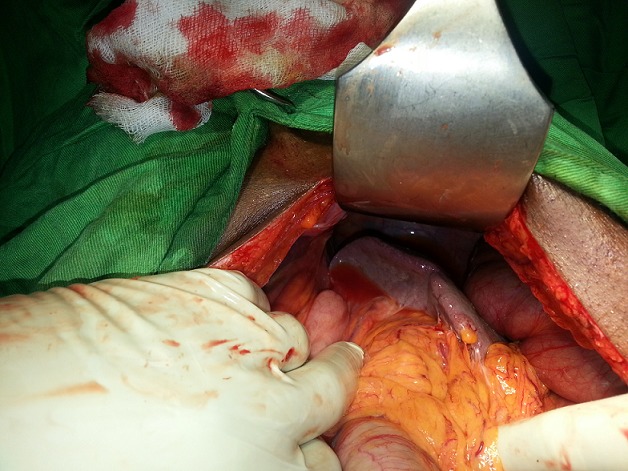
Rate et angle colique gauche ascensionnés avec la coupole diaphragmatique gauche

Un patient de 77 ans a été référé pour douleur abdominale et constipation évoluant 6 jours avant l'admission. Il y avait comme antécédents une hypertension artérielle et une hémiplégie gauche dans les suites d'un accident vasculaire cérébral il y a 27 ans. Il n'y avait pas d'antécédent de laparotomie, de traumatisme ni de manœuvres endoscopiques récentes. L'examen à l'admission avait permis de noter un patient apyrétique, une asthénie et des signes évidents de déshydratation. L'abdomen était légèrement météorisé, souple sans signes d'irritation péritonéale. Le toucher rectal était normal. L'examen du thorax était normal. Le compte de leucocytes et la CRP étaient normaux, respectivement 9300 GB/mm^3^ et 5mg/ml. La radiographie de l'abdomen sans préparation montrait un volumineux péritoine bilatéral contrastant avec l'absence de signes d'irritation péritonéale et infectieux biologiques (Figure 1). Les explorations ont été complétées par une TDM abdomino-pelvienne (Figure 2) qui a également mis en évidence un important pneumopéritoine, sans épanchement liquidien péritonéal et sans indices de perforation digestive. Un transit oeso-gastroduodénal (Figure 3) et un lavement hydrosolubles ont été également réalisés et ne montraient pas d'extravasion de produit de contraste. En l'absence d'arguments en faveur d'une perforation digestive, ni d'aucune autre cause, le diagnostic de pneumopéritoine spontané a été retenu. Un traitement médical conservateur a été institué et a consisté en une alimentation parentérale hyper-protidique hypercalorique avec une équilibration hydro-électrolytique. L'évolution a été favorable avec un maintien de l'apyrexie, l'amendement de la constipation et des douleurs à J3 d'hospitalisation. La sortie a été autorisée à J7 d'hospitalisation. Le contrôle à 1 mois était normal avec une radiographie de l'abdomen sans préparation qui ne notait plus de pneumopéritoine (Figure 4).

## Discussion

Les termes pneumopéritoine spontané (PS), pneumopéritoine non chirurgical et pneumopéritoine spontané idiopathique (PSI) peuvent prêter à confusion. Un pneumopéritoine est dit spontané lorsqu'il n'est pas associé à une perforation d'organe creux [2]. Il sera qualifié d'idiopathique au terme d'un bilan étiologique négatif, excluant d'autres causes de pneumopéritoine [3]. Le terme pneumopéritoine non chirurgical est commun aux deux situations qui autorisent généralement un traitement conservateur [1]. Le PS correspond à moins de 10% de l'ensemble des cas de pneumopéritoine [4]. La difficulté du diagnostic réside dans l'exclusion formelle d'une perforation viscérale sans exploration chirurgicale. Six patients sur 7 présentant un PS ont ainsi été opérés dans la série mono centrique de Van Gelder *et al* [5]. Lorsqu'il y a une symptomatologie abdominale comme chez notre patient, il faut prendre en compte l'absence de fièvre, d'irritation péritonéale et d'hyperleucocytose, signes habituellement absents dans les pneumopéritoines non chirurgicaux [2, 6]. Dans ces cas et particulièrement devant un patient fragile il est licite de surseoir à une intervention urgente et de faire la preuve préopératoire d'une perforation digestive. Cette preuve peut être obtenue par la TDM abdominale [6, 7]. Elle permet le diagnostic d'une perforation mais aussi de prédire précisément la localisation de la perforation avec une précision de 86% [7]. La TDM permet par ailleurs de rechercher une étiologie rare de pneumopéritoine spontanée, la pneumatose cystique intestinale [8]. Lorsqu'un PS est évoqué, l'enquête étiologique doit exploiter le contexte clinique. Le PS peut être observé au décours d'explorations endoscopiques ou encore dans des circonstances favorisant l'augmentation de la pression thoracique [2, 4]. Ces dernières sont celles qui sont le plus fréquemment en cause dans les PS [9]. Les patients ventilés sous pression positive, les barotraumatismes, les fistules trachéales, les manœuvres de réanimations cardio-respiratoires répondent à ce mécanisme. Le pneumopéritoine peut dans ce cas être associé à un pneumo médiastin ou à un pneumothorax [2]. Au terme de la démarche diagnostique il est possible qu'aucune cause ne soit retrouvée, même en présence d'un volumineux pneumopéritoine [6]. Chez ces patients, il faut choisir entre un risque important de réaliser une laparotomie inutile [1-3] et un risque raisonnablement réduit d'ignorer une affection chirurgicale en réalisant un traitement conservateur. Celui-ci a été efficace dans notre observation comme dans plusieurs autres dans la revue de Tanaka *et al* [6].

## Conclusion

L'existence d'un pneumopéritoine ne doit pas empêcher une démarche clinique et para-clinique méthodique pour réunir des arguments en faveur d'une perforation digestive. En l'absence de ces arguments, il faut savoir évoquer un pneumopéritoine spontané et surseoir à une intervention urgente.

## Conflits d’intérêts

Les auteurs ne déclarent aucun conflit d'intérêts.
